# A UK nationwide study of people with type 1 diabetes admitted to hospital with COVID-19 infection

**DOI:** 10.1007/s00125-021-05463-x

**Published:** 2021-05-08

**Authors:** Yue Ruan, Robert E. J. Ryder, Parijat De, Benjamin C. T. Field, Parth Narendran, Ahmed Iqbal, Rajiv Gandhi, Sophie Harris, Dinesh Nagi, Umaira Aziz, Efthimia Karra, Sandip Ghosh, Wasim Hanif, Amy E. Edwards, Mansoor Zafar, Umesh Dashora, Kinga A. Várnai, Jim Davies, Sarah H. Wild, Emma G. Wilmot, David Webb, Kamlesh Khunti, Rustam Rea, on behalf of the ABCD Covid-19 audit group

**Affiliations:** 1 Oxford Centre for Diabetes, Endocrinology and Metabolism, Oxford University Hospitals NHS Foundation Trust, Oxford, UK; 2 Oxford NIHR Biomedical Research Centre, Oxford, UK; 3 Sandwell and West Birmingham Hospitals NHS Trust, Birmingham, UK; 4 Department of Clinical & Experimental Medicine, Faculty of Health & Medical Sciences, University of Surrey, Guildford, UK; 5 Department of Diabetes & Endocrinology, Surrey & Sussex Healthcare NHS Trust, Redhill, Surrey, UK; 6 Medical and Dental Sciences, University of Birmingham, Birmingham, UK; 7 Diabetes Centre, The Queen Elizabeth Hospital, University Hospitals Birmingham NHS Foundation Trust, Birmingham, UK; 8 Department of Diabetes & Endocrinology, Sheffield Teaching Hospitals NHS Foundation Trust, Sheffield, UK; 9 Diabetes and Endocrinology Department, King’s College Hospital, London, UK; 10 Mid Yorkshire Hospitals NHS Trust, Pinderfields Hospital, Wakefield, UK; 11 Royal Free Hospital, London, UK; 12 Department of Diabetes and Endocrinology, Newham University Hospital, Barts Health NHS Trust, London, UK; 13 Conquest Hospital, Hastings, UK; 14 Oxford University Hospitals NHS Foundation Trust, Oxford, UK; 15 Department of Computer Science, University of Oxford, Oxford, UK; 16 Usher Institute, University of Edinburgh, Edinburgh, UK; 17 Diabetes Department, University Hospitals of Derby and Burton NHS FT, Derby, UK; 18 University of Nottingham, Nottingham, UK; 19 Diabetes Research Centre, University Hospitals of Leicester NHS Trust, Leicester General Hospital, Leicester, UK

**Keywords:** COVID-19, Inpatients, Mortality, National audit, Type 1 diabetes

## Abstract

**Aims/hypothesis:**

The aim of this work was to describe the clinical characteristics of adults with type 1 diabetes admitted to hospital and the risk factors associated with severe coronavirus disease-2019 (COVID-19) in the UK.

**Methods:**

A retrospective cohort study was performed using data collected through a nationwide audit of people admitted to hospital with diabetes and COVID-19, conducted by the Association of British Clinical Diabetologists from March to October 2020. Prespecified demographic, clinical, medication and laboratory data were collected from the electronic and paper medical record systems of the participating hospitals by local clinicians. The primary outcome of the study, severe COVID-19, was defined as death in hospital and/or admission to the adult intensive care unit (AICU). Logistic regression models were used to generate age-adjusted ORs.

**Results:**

Forty UK centres submitted data. The final dataset included 196 adults who were admitted to hospital and had both type 1 diabetes and COVID-19 on admission (male sex 55%, white 70%, with mean [SD] age 62 [19] years, BMI 28.3 [7.3] kg/m^2^ and last recorded HbA_1c_ 76 [31] mmol/mol [9.1 (5.0)%]). The prevalence of pre-existing microvascular disease and macrovascular disease was 56% and 39%, respectively. The prevalence of diabetic ketoacidosis on admission was 29%. A total of 68 patients (35%) died or were admitted to AICU. The proportions of people that died were 7%, 38% and 38% of those aged <55, 55–74 and ≥75 years, respectively. BMI, serum creatinine levels and having one or more microvascular complications were positively associated with the primary outcome after adjusting for age.

**Conclusions/interpretation:**

In people with type 1 diabetes and COVID-19 who were admitted to hospital in the UK, higher BMI, poorer renal function and presence of microvascular complications were associated with greater risk of death and/or admission to AICU. Risk of severe COVID-19 is reassuringly very low in people with type 1 diabetes who are under 55 years of age without microvascular or macrovascular disease.

**In people with Type 1 diabetes and COVID-19 admitted to hospital in the UK, BMI and one or more microvascular complications had a positive association and low serum creatine levels had a negative association with death/admission to intensive care unit after adjusting for age.:**

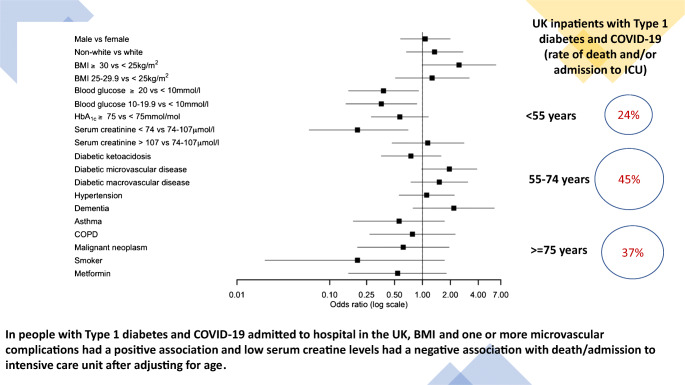

**Supplementary Information:**

The online version contains peer-reviewed but unedited supplementary material available at 10.1007/s00125-021-05463-x.

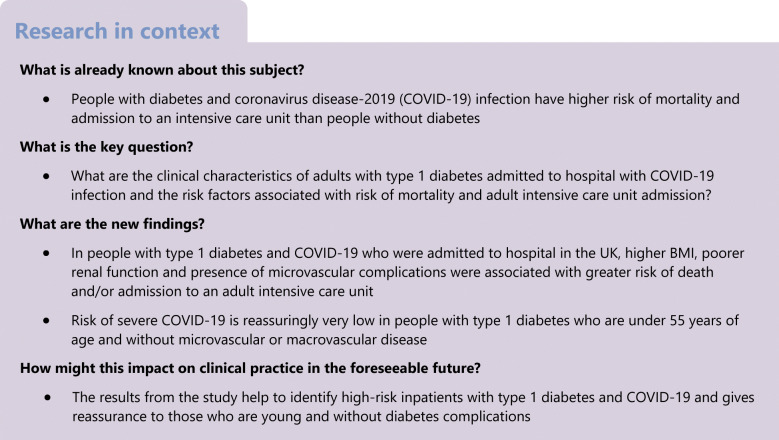



## Introduction

The severe acute respiratory syndrome coronavirus-2 (SARS-CoV-2) was first reported in Wuhan, China in December 2019 and has since been responsible for over 1.6 million deaths from coronavirus disease-2019 (COVID-19) infection globally by December 2020 [1]. A number of studies and systematic reviews have reported that certain chronic comorbidities, such as hypertension, diabetes, CVD, chronic kidney disease and chronic obstructive pulmonary disease, are associated with increased risk of severe COVID-19 and mortality [2]. Within the population of 61 million people in England, approximately 0.4% have type 1 diabetes and 4.7% have type 2 diabetes. An English population-based study reported that, up to 11 May 2020, 23,698 in-hospital COVID-19-related deaths had occurred, of which 1.5% were people with type 1 diabetes and 31.4% were people with type 2 diabetes [3]. The study estimated that ORs for in-hospital COVID-19-related death (adjusted for age, sex, deprivation, ethnicity and geographical region) was 3.51 (95% CI 3.16, 3.90) for type 1 diabetes and 2.03 (1.97, 2.09) for type 2 diabetes compared with people without diabetes [3].

Several studies have also reported the association between admission hyperglycaemia and in-hospital mortality rate in people with diabetes. An Italian study of 59 hospitalised patients with COVID-19 showed that those with hyperglycaemia or diabetes had a higher mortality rate compared with those without diabetes and euglycaemia [4]. Newly diagnosed diabetes was associated with an increased mortality rate in a study of 453 patients admitted to hospital with COVID-19 in Wuhan, China (HR compared with people with normal glucose levels 9.42 [95% CI 2.18, 40.7]) [5]. A further study of 605 COVID-19 patients admitted to two hospitals in Wuhan showed that fasting plasma glucose >7.0 mmol/l was independently associated with higher 28 day mortality rate after adjustment for age, sex and a measure of COVID-19 disease severity (HR 2.30 [95% CI 1.49, 3.55]) [6]. All these studies suggest that hyperglycaemia, including newly diagnosed diabetes, in people with COVID-19 is associated with higher rates of complications and mortality. However, most studies have been small, from single centres or single cities, and have not examined the complications and mortality outcomes in a specific subset of people with diabetes. Few studies have had sufficient power to describe factors associated with poor outcomes among people with type 1 diabetes [7–9]. It is important to gain further insights into the characteristics of people with type 1 diabetes and severe COVID-19 in order to inform clinical practice and provide accurate information to people with type 1 diabetes. The aims of this study are to describe the characteristics of people with type 1 diabetes admitted to hospital with COVID-19 and to identify the risk factors associated with severe disease.

## Methods

### Data collection

Data for this retrospective cohort study were collected through a nationwide audit conducted by the Association of British Clinical Diabetologists (ABCD) [10]. A full list of ABCD Collaborators can be found in the electronic supplementary material (ESM). The nationwide audit commenced in September 2020. Contributors were asked to collate data from patient records and to transfer the data in anonymous form to the National Institute for Health Research (NIHR) Health Informatics Collaborative (HIC) Coordinating Centre within the Oxford University Hospitals NHS Foundation Trust (OUH). The data were transferred securely using the National Health Service (NHS) network. Submissions were checked by the NIHR HIC team and additional information was sought from contributing centres where necessary to ensure completeness and accuracy. The data were processed and analysed on a secure server within OUH.

### Ethical approval

The audit was registered with the OUH and a Data Protection Impact Assessment was carried out and approved by the OUH Caldicott Guardian and the Public Benefit and Privacy Panel in Scotland (reference 2021–0111). The NHS supports audit with clear guidance for the contributing centres on the use of routine clinical practice data submitted in anonymised form via the secure NHS network. As the study was retrospective, and comprised routinely collected healthcare data only, there was no requirement for approval by a research ethics committee.

### Study variables

Inpatients’ demographic information, clinical characteristics, medication history and laboratory measurements were collected from the electronic and paper medical record systems of the participating centres by local clinicians using a standard template. Demographic data comprised age in years, sex, ethnicity and census-derived Index of Multiple Deprivation decile. The following clinical characteristics were included: weight and height, or BMI; classification of diabetes; duration of diabetes; diabetes complications including diabetic ketoacidosis (DKA), diabetic foot ulcer, diabetic nephropathy, diabetic peripheral neuropathy, diabetic retinopathy, peripheral vascular disease, ischaemic heart disease (myocardial infarction) and/or heart failure, and cerebrovascular disease (stroke/transient ischaemic attack); and other significant comorbidities (hypertension, dementia, asthma, chronic obstructive pulmonary disease, malignant neoplasm, smoking status). Medication history included glucose-lowering medications and other selected drug classes (angiotensin-converting enzyme inhibitors or angiotensin receptor antagonists, oral corticosteroids, statins, antiplatelet agents, anticoagulant agents and regular nonsteroidal anti-inflammatory drugs). Laboratory data included latest pre-admission HbA_1c_ and serum creatinine, and admission assays of blood glucose, pH, bicarbonate, lactate, serum creatinine and capillary blood ketones. Dates of the start and finish (if applicable) of each hospital admission were collected, along with the date of positive SARS-CoV-2 test (a positive result was a prerequisite for inclusion in the study). Recorded outcomes included vital status and admission to an adult intensive care unit (AICU) during the entire hospital admission. The variables relevant to the analysis of the type 1 diabetes population were used and further data processing and analysis is ongoing for the type 2 diabetes population. The ABCD audit remains open and the original data collection sheet can be obtained from the ABCD secretariat.

### Statistical analysis

This report is based on people with type 1 diabetes only. Baseline clinical characteristics are reported as frequency and percentages for categorical variables, and as mean and SD for continuous variables. The primary outcome of the study was severe disease, defined as death or admission to the AICU. Mortality rate alone was a secondary outcome. Logistic regression models, with adjustment for age as a continuous variable, were used to generate ORs for the primary outcome. For the logistic regression analysis, continuous variables, including BMI, admission blood glucose, latest HbA_1c_ and serum creatinine, were transformed into categorical variables (BMI <25 [reference category], 25–29.9 and ≥30 kg/m^2^; admission blood glucose <10 [reference category], 10–19.9 and ≥20 mmol/l; HbA_1c_ < 75 [reference category] and ≥75 mmol/mol; serum creatinine <74, 74–107 [reference category, regarded as the normal range] and >107 μmol/l). Binary categorical variables were also created to define sex (1 for male vs 0 for female), ethnicity (1 for non-white vs 0 for white), DKA (1 for yes), hypertension (1 for yes), dementia (1 for yes), asthma (1 for yes), chronic obstructive pulmonary disease (1 for yes), malignant neoplasm (1 for yes), smoking status (1 for current/previous smoker, 0 for never smoker), metformin use (1 for yes, 0 for no metformin), microvascular disease (1 for having one or more conditions from diabetic foot ulcer, diabetic nephropathy, diabetic peripheral neuropathy and diabetic retinopathy) and macrovascular disease (1 for having one or more conditions from peripheral vascular disease, ischaemic heart disease and cerebrovascular disease). All statistical analysis was performed using R version 3.3 (www.r-project.org/). A *p* value of <0.05 was considered statistically significant.

## Results

### Demographic and clinical characteristics

Up to 8 December 2020, a total of 40 NHS centres around the UK had submitted data to the ABCD COVID-19 diabetes national audit. The final dataset consisted of a total of 3312 patient records; 196 were for inpatients with type 1 diabetes who were hospitalised between March and October 2020, of whom 194 patients had primary outcome data submitted (discharged or died or admitted to ICU). All patients were classified by the local clinical teams as having type 1 diabetes and were confirmed to be receiving insulin therapy.

Table 1 summarises the clinical characteristics of the whole study population stratified by the primary outcome. There was a slightly higher proportion of men (55%), mean (SD) age was 62 (19) years and BMI was 28.3 (7.3) kg/m^2^, and the majority of people were of white ethnicity (70%). The mean (SD) latest available HbA_1c_ was 76 (31) mmol/mol (9.1 [5.0]%). In 80 patients (50%), the latest available HbA_1c_ was <58 mmol/mol (<7.5%). The prevalence of diabetic retinopathy was 45% and of hypertension was 59%. Overall, 56% of people had a microvascular complication (reported diabetic nephropathy, foot ulcer, retinopathy, peripheral neuropathy) and 39% a macrovascular complication (reported peripheral vascular disease, ischaemic heart disease, cerebrovascular disease).

**Table 1:** Clinical characteristics of the type 1 diabetes population in the ABCD COVID-19 audit

Clinical features	Available data (*N* = 196)	All	Death and/or AICU	
			Yes	No
Male sex	195	108/195 (55)	38/67 (57)	70/128 (55)
Age, years	194	62 ± 19	65 ± 16	56 ± 21
Ethnicity	156			
White		109/156 (70)	42/62 (68)	65/94 (69)
Asian		12/156 (8)	4/62 (6)	8/94 (9)
Black		20/156 (13)	12/62 (19)	8/94 (9)
Other		15/156 (10)	4/62 (6)	13/94 (14)
BMI, kg/m^2^	121	28.3 ± 7.3	28.9 ± 7.7	27.8 ± 7.0
Admission blood glucose, mmol/l	122	18.6 ± 11.3	17.3 ± 13.5	19.6 ± 10.2
HbA_1c_, mmol/mol	161	76 ± 31	66 ± 26	80 ± 33
HbA_1c_, %	161	9.1 ± 5.0	8.2 ± 4.5	9.5 ± 5.2
Creatinine, μmol/l	137	142 ± 81	181 ± 93	116 ± 71
Diabetes complications				
DKA on admission	171	49/171 (29)	14/64 (22)	35/107 (33)
Diabetic foot ulcer	149	32/149 (21)	12/54 (22)	20/95 (21)
Diabetic nephropathy	128	43/128 (34)	20/50 (40)	23/78 (29)
Diabetic peripheral neuropathy	140	48/140 (34)	21/55 (38)	27/85 (32)
Diabetic retinopathy	139	63/139 (45)	27/54 (50)	36/85 (42)
Microvascular disease^a^	176	98/176 (56)	45/65 (69)	53/111 (48)
Macrovascular disease^b^	156	61/156 (39)	27/46 (59)	34/110 (31)
Comorbidities				
Hypertension	167	98/167 (59)	40/65 (62)	58/102 (57)
Dementia	160	22/160 (14)	13/55 (24)	9/105 (9)
Asthma	150	21/150 (14)	5/61 (8)	16/89 (18)
COPD	149	17/149 (11)	7/61 (11)	10/88 (11)
Malignant neoplasm	154	15/154 (10)	5/61 (8)	10/93 (11)
Treatment on admission				
Metformin	161	19/161 (12)	4/62 (6)	15/99 (15)
Insulin pump	106	9/106 (8)	1/45 (2)	8/61 (13)
Smoker	100	9/100 (9)	1/37 (3)	8/63 (13)

Data are shown as mean ± SD or *n*/total *n* (%), unless stated otherwise

^a^Microvascular disease includes diabetic nephropathy, foot ulcer, retinopathy and peripheral neuropathy

^b^Macrovascular disease includes peripheral vascular disease, ischaemic heart disease and cerebrovascular disease

COPD, chronic obstructive pulmonary disease

Table 2 describes the clinical outcomes in the whole type 1 diabetes population and in three different age groups. Twenty-seven per cent of the overall population died from COVID-19 (53/194) and there was a marked age gradient, with 7%, 38% and 38% of people aged <55, 55–74 and ≥75 years dying, respectively. ESM Fig. 1 plots the distribution of admission durations for those patients who died in hospital. The median (IQR) time between admission and hospital death was 7 [4, 15] days. Most deaths (30 out of 53) occurred within 1 week after admission but two deaths occurred after 7 weeks. A total of 68 patients (35%) died and/or were admitted to the AICU.

**Table 2:** Outcomes in patients with type 1 diabetes and COVID-19 in different age groups

Outcome	Available data (*N* = 196)	All age groups	<55 years	55–74 years	≥75 years
Death, *n*/total *n* (%)	194	53/194 (27)	5/67 (7)	29/77 (38)	19/50 (38)
Death and/or AICU, *n*/total *n* (%)	194	68/194 (35)	17/72 (24)	32/71 (45)	19/51 (37)

### Logistic regression analysis

**Fig. 1:**
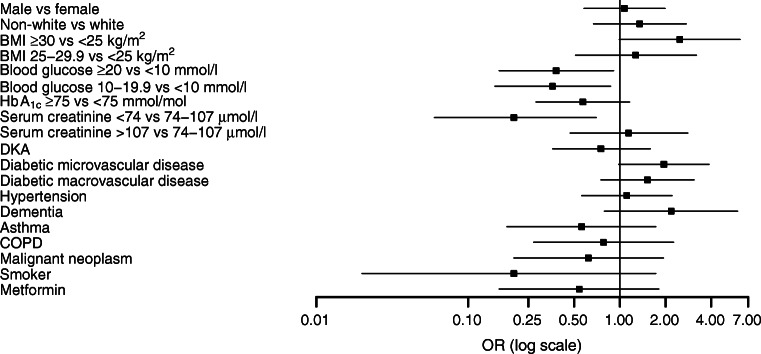
Forest plot of the ORs (95% CIs) for the association between severe COVID-19 (death or AICU admission) and clinical variables after adjusting for age. The comparison groups (sample size) are: male vs female (*n* = 195); non-white vs white (*n* = 156); BMI ≥30 kg/m^2^ vs <25 kg/m^2^ (*n* = 121); BMI 25–29.9 kg/m^2^ vs <25 kg/m^2^ (*n* = 121); blood glucose ≥20 mmol/l vs <10 mmol/l (*n* = 122); blood glucose 10–19.9 mmol/l vs <10 mmol/l (*n* = 122); HbA1c ≥75 mmol/mol (≥9.0%) vs <75 mmol/mol (<9.0%) (*n* = 161); serum creatinine <74 μmol/l vs 74–107 μmol/l (*n* = 137); serum creatinine >107 μmol/l vs 74–107 μmol/l (*n* = 137); DKA vs no DKA (*n* = 171); diabetic microvascular disease vs no diabetic microvascular disease (*n* = 176); diabetic macrovascular disease vs no diabetic macrovascular disease (*n* = 156); hypertension vs no hypertension (*n* = 167); dementia vs no dementia (*n* = 160); asthma vs no asthma (*n* = 150), chronic obstructive pulmonary disease (COPD) vs no COPD (*n* = 149); malignant neoplasm vs no malignant neoplasm (*n* = 154); smoker vs non-smoker (*n* = 100); metformin vs no metformin (*n* = 161). The base used for logarithmic transformations on the *x*-axis is log_10_

Figure 1 shows a forest plot with ORs and 95% CIs of death and/or AICU admission for different clinical variables after adjusting for age. ESM Table 1 reports the ORs for the primary outcome and ESM Table 2 reports the ORs for mortality rate alone. BMI, serum creatinine and presence of microvascular disease were positively associated with the primary outcome after adjusting for age while, surprisingly, higher admission blood glucose levels were negatively associated with the primary outcome after age adjustment. However, there was a statistically significant difference in the mean blood glucose value between people with DKA and those without DKA which is likely to account for this unexpected finding. In people with DKA, the mean (SD) admission blood glucose level was 29 (14) mmol/l, while in people without DKA it was 15 ± 7 mmol/l (*p* < 0.05).

Serum creatinine and presence of macrovascular disease were positively associated with mortality rate alone after adjusting for age (ESM Table 2).

ESM Fig. 2 shows the distribution of eGFR, with the majority of patients having abnormal pre-admission renal function (eGFR <90 ml min^−1^ [1.73 m]^−2^).

## Discussion

To the best of our knowledge, this study provides an analysis of the largest cohort yet assembled of people with type 1 diabetes admitted to hospital with COVID-19 infection for whom detailed clinical information is available. We report distributions of age, sex, ethnicity, diabetic complications, comorbidities, treatment on admission and laboratory test results, stratified by outcome.

The results from the present study gives reassurance to the younger type 1 diabetes population without diabetes complications with regards to their risk of severe COVID-19. However, five out of 67 (7%) patients aged <55 years old died. Of these five people, three were admitted to hospital primarily for COVID-19 and two for DKA and all of them had one or both of microvascular and macrovascular complications. There were no deaths in people with type 1 diabetes under the age of 55 years old without diabetic complications.

Overall, 49 patients were admitted to hospital with DKA (29% of 171 patients for whom data were available). This compares with 7.3% of patients who were admitted to hospital specifically for the management of diabetes in the 2019 UK National Diabetes Inpatient Audit [11]. This fourfold increase in DKA requiring hospital admission in people with type 1 diabetes is a significant concern both for the population as well as the healthcare system. Our findings emphasise the importance of supporting people with type 1 diabetes in the community during the pandemic, to minimise the incidence of avoidable admission. Health policy development may require population-specific attention to ethnicity as a risk factor for DKA, because DKA incidence varied fourfold between ethnic groups in a recently published US study [12]. We performed a further analysis to re-examine the association between blood glucose levels and the primary outcome after adjusting for both age and DKA. The results show a non-significant association with the primary outcome for blood glucose ≥20 mmol/l vs <10 mmol/l (OR 0.37 [95% CI 0.14, 1.03], *p* = 0.06) but the association did reach statistical significance when comparing blood glucose 10–19.9 mmol/l vs <10 mmol/l (OR 0.35 [95% CI 0.14, 0.89], *p* = 0.03). We excluded four patients who were admitted to hospital with blood glucose <4 mmol/l in this analysis. This unexpected finding of reduction in primary outcome (rate of death and AICU admission) in people with modestly elevated admission glucose compared with those with normal admission glucose needs further confirmation from a larger dataset.

We compared the clinical characteristics of this inpatient cohort with those of the general type 1 diabetes population included in the retrospective registry-based population study recently reported by the National Diabetes Audit (NDA) for England & Wales [13]. We found similar sex and ethnicity distribution (male, 55% vs 56%; white ethnicity, 70% vs 80%) but our cohort was of older age and relatively poor glycaemic control (proportion of age > 64 years, 45% vs 15%; HbA_1c_ ≤ 75 mmol/mol [9.0%], 40% vs 71%) in COVID-19 inpatients compared with the general type 1 diabetes population. The crude (i.e. not adjusted for age) prevalence of diabetes-related complications was higher in COVID-19 inpatients than in the general type 1 diabetes population as reported in the 2017–2018 data report [14]: diabetic foot ulcer, 21% vs 5%; ischaemic heart disease, 24% vs 1.9%; cerebrovascular disease, 15% vs 0.5%. The complications data in the NDA report are extracted from primary care data which may be up to 15 months old, whereas our findings are based on contemporaneous admission records.

To our knowledge, few studies have been published on characteristics or outcomes in people with type 1 diabetes and COVID-19. The largest study to date has been the population-based study in England (population 61 million people) describing in-hospital mortality rate among 364 people with type 1 diabetes (1.45% of all hospital admissions due to COVID-19) [3]. The study showed that mortality was mainly confined to an older population, with no deaths occurring in those aged <50 years [3]. Another UK population study linked data between the NDA and the Office of National Statistics and compared the mortality rate between the year 2020 and the three previous years [15]. The study found that weekly death registrations in the first 19 weeks of 2020 exceeded the corresponding 3 year weekly averages for 2017–2019 by 50.9% in people with type 1 diabetes and that the increased COVID-19-related mortality rate was associated with cardiovascular/renal complications of diabetes and with glycaemic control and BMI [15]. A recent Scottish population study [16] included 51 (0.1%) of 34,383 people with type 1 diabetes who developed fatal or critical care unit-treated COVID-19 between 1 March 2020 and 31 July 2020. Overall, the risk of fatal or critical care unit-treated COVID-19 was increased by 2.4 times in those with type 1 diabetes compared with those without diabetes. Previous hospital admissions with hypoglycaemia or DKA were strongly associated with fatal or critical care unit-treated COVD-19. Compared with the general population of people with type 1 diabetes, those in the fatal/AICU admission group were older (mean age 71.4 vs 44.5 years), had higher mean BMI (27 vs 26 kg/m^2^), higher mean HbA_1c_ (69 vs 67 mmol/mol), lower mean eGFR (72 vs 100 ml min^−1^ [1.73 m]^−2^), higher prevalence of heart disease (61% vs 14%) and lower insulin pump use (2% vs 14%). The patients included in this current study have not been excluded from other UK-based COVID registry studies. The present study included hospital admissions with type 1 diabetes and COVID from 1 March until 31 October 2020.

The Coronavirus-SARS-CoV-2 and Diabetes Outcomes (CORONADO) observational study from 68 French hospitals reported data for 56 people with type 1 diabetes [8]. In this cohort 55.4% were men, mean age was 56.0 years and mean BMI of 25.8 kg/m^2^. At 7 days, 11 patients (19.6%) had required tracheal intubation for mechanical ventilation, three had died (5.4%) and nine (16.1%) had been discharged. Overall, those with severe disease or who died were older (65.3 vs 53.2 years) and more likely to have hypertension (OR 5.21 [95% CI 1.24, 21.9]) than people who had not developed these outcomes within 7 days. Poor outcomes from COVID-19 in people with type 1 diabetes in this cohort were most strongly related to age, with no deaths occurring in those aged <65 years [8], whereas in a larger cohort with follow-up until discharge and a different healthcare system, we found a 7% mortality rate in people aged <55 years. The T1D exchange quality improvement collaborative (TIDX-QI), involving 64 sites in the USA, reported data on 33 patients with type 1 diabetes and laboratory-confirmed COVID-19 [7]. Among this study population, 54.5% were female and their mean age was 24.8 years and mean HbA_1c_ was 69 mmol/mol (8.5%) [7]. The most prevalent comorbidities reported in this study were obesity (39.4%) and hypertension or CVD (12.1%). DKA occurred in 45.5% of patients [7].

Another US study conducted a retrospective chart review in 35 people with type 1 diabetes admitted to the Beth Israel Deaconess Medical Centre in Boston, MA, of which seven had COVID-19 [9]. Although the cohort was small, the study indicated that, compared with those who were COVID-19 negative, a higher proportion of SARS-CoV-2-positive patients were of non-Hispanic Black American ethnicity, with no significant differences in sex, body weight, glucose or HbA_1c_ on admission. Only one patient in the COVID-19-positive group and two people in the COVID-19-negative group had DKA. The study showed similar glycaemic control prior to admission among people with type 1 diabetes, regardless of COVID-19 test results [9].

Our study has several strengths. The data were collected from a large number of centres across the UK, using a structured proforma with variables prespecified based on previous studies. This is the largest published cohort of people with type 1 diabetes for whom contemporaneous admission data are available. The data include ethnicity and outcomes to discharge or death, up to 70 days from admission, reflecting the prolonged hospital course of many patients with severe COVID-19. Limitations to our study include the retrospective nature of data collection and the absence of data from contemporaneous hospital admissions of people with type 1 diabetes without COVID-19 infection, and from people with type 1 diabetes and COVID-19 who were not admitted to hospital. We recognise that the patient population admitted to hospital are significantly different from the wider type 1 diabetes population and so the conclusions should be restricted to this subgroup of people. A number of centres had missing data for some of the variables (we report the completeness of variables in Table 1).

In conclusion, we report the largest study of people with type 1 diabetes admitted to hospital with COVID-19. In this population, higher BMI, worse renal function and the presence of microvascular complications were associated with higher risk of death and/or admission to AICU. However, no people with type 1 diabetes <55 years of age without microvascular or macrovascular complications died or were admitted to AICU.

## Supplementary Information

ESM 1 (PDF 219 kb)

## Data Availability

The ABCD audit remains open and the original data collection sheet can be obtained from the ABCD secretariat. Data are available on request from the authors.

## Abbreviations

ABCD: Association of British Clinical Diabetologists
AICU: Adult intensive care unit
COVID-19: Coronavirus disease-2019
DKA: Diabetic ketoacidosis
NDA: National Diabetes Audit
NHS: National Health Service
NIHR: National Institute for Health Research
OUH: Oxford University Hospitals NHS Foundation Trust
SARS-CoV-2: Severe acute respiratory syndrome coronavairus-2
