# Supplementary material for: A UK nationwide study of people with type 1 diabetes admitted to hospital with COVID-19 infection
Source: Diabetologia. 2021 May 8;64(8):1717–24. doi: 10.1007/s00125-021-05463-x (PMC8106514; doi:10.1007/s00125-021-05463-x)
Supplement: Supplementary file 1 — (PDF 219 kb) [file 125_2021_5463_MOESM1_ESM.pdf]

Electronic supplementary material

ESM Table 1. Odds-ratios with 95% confidence intervals of death and/or AICU for different clinical variables after adjusting for age (see Table 1 for sample sizes for each variable)

Clinical features	OR (95% CI)	*p* value
Sex	Male vs female 1.07(0.58-1.99)	0.82
Ethnicity	Non-white vs white 1.35(0.67-2.74)	0.38
BMI	Per kg/m2 1.03(0.98-1.08)<br>BMI>=30 vs <25 **2.48(1.00-6.20)**<br>BMI 25-29.9 vs< 25 1.27(0.51-3.20)	0.30<br>**0.05**<br>0.32
Admission blood glucose	Per mmol/l 0.98(0.95-1.02)<br>BG>=20 vs <10 **0.38(0.16-0.91)**<br>BG 10-19.9 vs <10 **0.36(0.15-0.87)**	0.34<br>**0.04**<br>**0.04**
HbA1c (mmol/mol(%))	Per mmol/mol 0.99(0.98-1.00)<br>HbA1c >=75(9%) vs <75(9%) 0.57(0.28-1.16)	0.06<br>0.12
Creatinine	Per unit **1.01(1.01-1.01)**<br>Creatinine <74 vs 74-107 0.20(0.06-0.70)<br>Creatinine >107 vs 74-107 1.14(0.47-2.81)	**6.2e-5**<br>**0.002**<br>0.21
**Diabetes Complications**		
DKA on admission	0.75(0.36-1.59)	0.46
Diabetic microvascular disease (Diabetic nephropathy, foot ulcer, retinopathy, peripheral neuropathy)	1.95(1.00-3.87)	**0.05**
Diabetic macrovascular disease (Peripheral vascular disease, Ischaemic heart disease and Cerebrovascular disease)	1.52(0.75-3.09)	0.35
**Comorbidities**		
Hypertension	1.11(0.56-2.21)	0.77
Dementia	2.18(0.79-5.97)	0.13
Asthma	0.56(0.18-1.73)	0.32
COPD	0.78(0.27-2.26)	0.65
Malignant neoplasm	0.62(0.20-1.94)	0.41
**Treatment on admission**		
Metformin	0.54(0.16-1.81)	0.32
ACE-I/ARB	1.57(0.77-3.23)	0.21

ESM Table 2. Odds-ratios with 95% confidence intervals of death alone for different clinical variables after adjusting for age.

**Clinical features**	**OR (95% CI)**	***p* value**
Sex	Male vs female 1.45(0.71-2.96)	0.31
Ethnicity	Non-white vs white 1.21(0.54-2.67)	0.64
BMI	BMI>=30 vs <25 1.41(0.52-3.87)	0.30
	BMI 25-29.9 vs< 25 1.15(0.42-3.15)	0.79
Admission blood glucose	BG>=20 vs <10 0.61(0.23-1.66)	0.34
	BG 10-19.9 vs <10 0.75(0.29-1.96)	0.56
HbA1c (mmol/mol(%))	HbA1c >=75(9%) vs <75(9%) 0.67(0.29-1.51)	0.33
Creatinine	Creatinine <74 vs 74-107 0.25(0.07-0.89)	**0.04**
	Creatinine >107 vs 74-107 0.82(0.29-2.32)	0.27
**Diabetes Complications**		
DKA on admission	0.63(0.24-1.69)	0.36
Diabetic microvascular disease (Diabetic nephropathy, foot ulcer, retinopathy, peripheral neuropathy)	1.53(0.68-3.41)	0.20
Diabetic macrovascular disease (Peripheral vascular disease, Ischaemic heart disease and Cerebrovascular disease)	2.37(1.09-5.16)	**0.02**
**Comorbidities**		
Hypertension	0.88(0.41-1.86)	0.73
Dementia	2.34(0.81-6.77)	0.11
Asthma	0.40(0.08-1.96)	0.26
COPD	0.59(0.19-1.80)	0.35
Malignant neoplasm	0.52(0.15-1.86)	0.32
**Treatment on admission**		
Metformin	1.05(0.08-13.23)	0.88
ACE-I/ARB	1.59(0.71-3.56)	0.26

ESM Figure 1. Distribution of number of T1D deaths over time since admission to hospital.

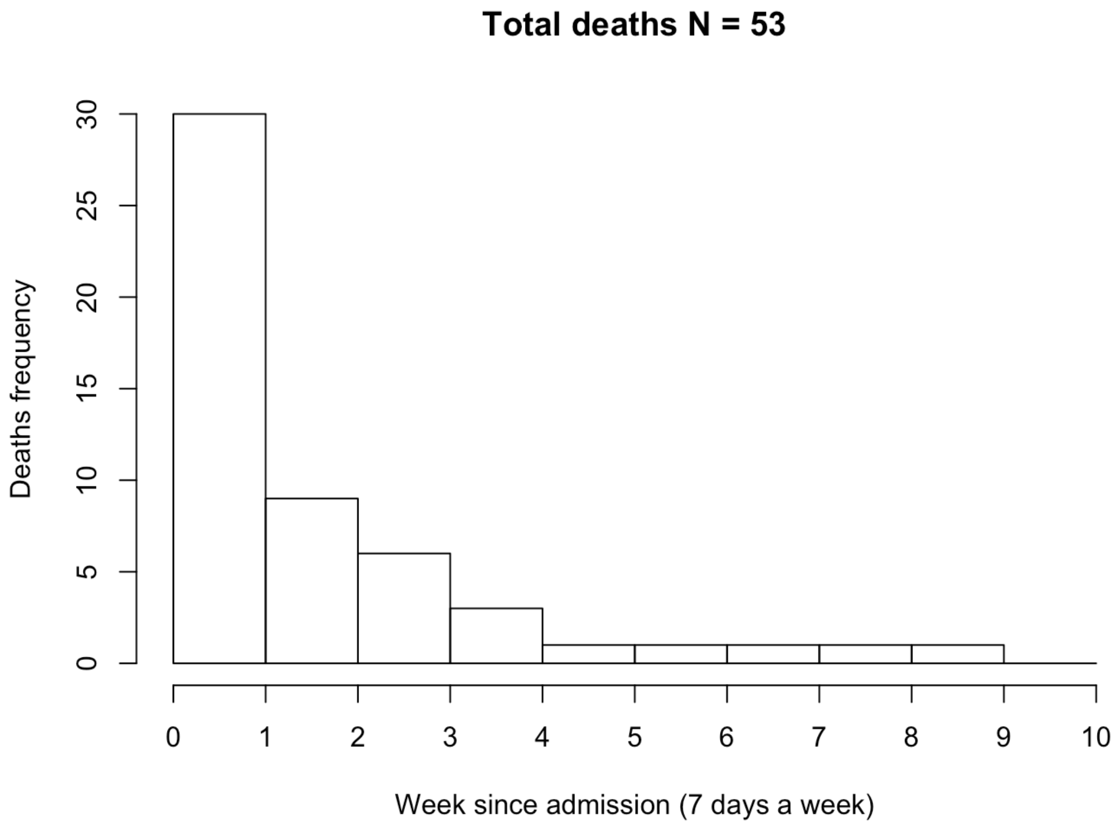

ESM Figure 2. Distribution of eGFR categories in the T1D population (eGFR calculated from pre-admission creatinine levels using the following formula: eGFR from the MDRD equation = 186 x $(\text{Creatinine}/88.4)^{-1.154}$ x $(\text{Age})^{-0.203}$ x (0.742 if female) x (1.210 if black)).

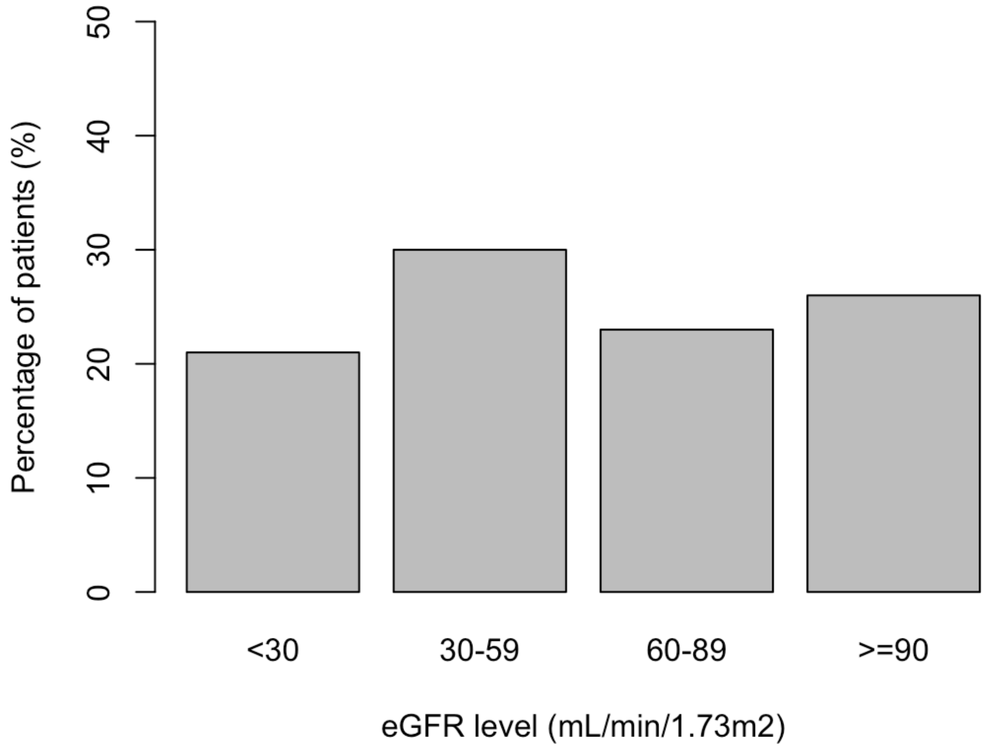

Collaborators

England

Barts Health NHS Trust, Newham University Hospital, London, UK: Amy Edwards; Susan Gelding; Kirun Gunganah.
Brighton and Sussex University Hospitals NHS Trust, Royal Sussex County Hospital, Brighton, UK: Ali Chakera; Dominique Rouse.
Dartford and Gravesham NHS Trust, Darent Valley Hospital, Dartford, UK: Amir Hayat; Cynthia Mohandas.
East and North Hertfordshire NHS Trust, Lister Hospital, Stevenage, UK: Htet Aung; Su Khant Chel; Nyan Lin; Kavitia Narula; Furruq Quadri; Su Lei Yin; Yin Yin.
East Kent Hospitals University NHS Foundation Trust, William Harvey Hospital, Ashford, UK: Alamin Alkundi; Abdelmajid Musa.
East Suffolk and North Essex NHS Foundation Trust, Colchester General Hospital, Colchester, UK: Emma Birbeck; Charles Bodmer.
East Sussex Healthcare NHS Trust, Conquest Hospital & Eastbourne District General Hospital, Hastings, UK: Irene Bossman; Sathis Kumar; Umesh Dashora; Elizabeth Toubi; Mansoor Zafar.
George Eliot Hospital NHS Trust, Diabetes Care Team, George Eliot Hospital, Nuneaton, UK: Vinod Patel; Amitha Gopinath.
Hull University Teaching Hospitals NHS Foundation Trust, Hull Royal Infirmary, Hull, UK: Belinda Allan.
King’s College Hospital NHS Foundation Trust, King’s College Hospital, London, UK: Dharshana Appuhamillage; Khubaib Ayoub; Sophie Harris; Charmaine Ilangaratne; Maliha Iqbal; Rory Maclean; Omar Mustafa.
Lancashire Teaching Hospitals NHS Foundation Trust, Royal Preston Hospital, Preston, UK: Susan Baxter.
London North West University Healthcare NHS Trust, Ealing Hospital, London, UK: Malgorzata Adamus; Kevin Baynes.
Maidstone and Tunbridge Wells NHS Trust, Maidstone Hospital, Maidstone, UK: Siva Sivappriyan.
Mid Yorkshire Hospitals NHS Trust, Pinderfields Hospital, Wakefield, UK: Ryan D’Costa; Dinesh Nagi.
North Bristol NHS Trust, Southmead Hospital, Bristol, UK: Vernon Parfitt.
North West Anglia NHS Foundation Trust, Hinchingbrooke Hospital, Huntingdon, UK: Sadia Nasir.
Oxford University Hospitals NHS Foundation Trust, John Radcliffe Hospital, Oxford, UK: Rustam Rea; Gail Roadknight; Kinga Várnai; Senthil Vasan.
Pennine Acute Hospitals NHS Trust, Diabetes Centre, North Manchester General Hospital, Manchester, UK: Vilashini Arul Devah.
Royal Berkshire NHS Foundation Trust (Berkshire Healthcare NHS Foundation Trust), Centre for Diabetes & Endocrinology, Reading, UK: Foteini Kavvoura.
Royal Devon and Exeter NHS Foundation Trust, Royal Devon and Exeter Hospital (Wonford), Exeter, UK: Lina Ficken; James Gilham; Vincent Simpson; Neil Walker.
Royal Free London NHS Foundation Trust, Royal Free Hospital, London, UK: Miranda Rosenthal; Efthimia Karra.
Salford Royal NHS Foundation Trust, Salford Royal Hospital, Salford, UK: Tracy Curran; Angela Paisley.
Sandwell & West Birmingham NHS Trust, City Hospital, Birmingham, UK: Melissa Cull; Parijat De; Priscilla Sarker; Robert Ryder.

Sheffield Teaching Hospitals NHS Foundation Trust, Northern General Hospital, Sheffield, UK: Rajiv Gandi.
Surrey & Sussex Healthcare NHS Trust, East Surrey Hospital, Redhill, UK: James Clark; Vesna Hogan; Lauren Jackson; Jamie-Leigh Williamson; Younes R. Younes.
The Newcastle Upon Tyne Hospitals NHS Foundation Trust, The Royal Victoria Infirmary, Newcastle Upon Tyne, UK: Lydia Grixti; Suann Tee.
University Hospital of Derby and Burton NHS Foundation Trust, Royal Derby Hospital and Queen's Hospital Burton, Burton-on-Trent, UK: Abilash Sathya; Emma Wilmot.
University Hospital Southampton NHS Foundation Trust, Diabetes at UHSNHSFT, Southampton, UK: Mayank Patel.
University Hospitals Birmingham NHS Foundation Trust, Heartlands Hospital, Birmingham, UK: Catherine Holmes.
University Hospitals Birmingham NHS Foundation Trust, University Hospital of Birmingham, Birmingham, UK: Wasim Hanif; Sandip Ghosh; Parth Narendran.
University Hospitals of Leicester NHS Trust, Diabetes Research Centre, Leicester General Hospital, Leicester, UK: Ehtasham Ahmad; Ejaz Ahmed, Melanie Davies; Kamlesh Khunti; David Webb.
University of Leicester, Leicester, UK: Ben Maylor.
University of Oxford, Oxford, UK: Jim Davies; Oliver Freeman; Steve Harris; Yue Ruan.
West Suffolk Hospital NHS Foundation Trust, West Suffolk Hospital, Bury Saint Edmunds, UK: Anupam Brahma.
Yeovil District Hospital NHS Foundation Trust, Yeovil District Hospital, Yeovil, UK: Seshadri Pramodh.

Scotland
NHS Forth Valley, Forth Valley Royal Hospital, Larbert, UK: Katy Frew; Alison Mackenzie; Abigail Wild.
NHS Greater Glasgow and Clyde, Queen Elizabeth University Hospital, Glasgow, UK: Helen Casey; Deborah Morrison; Conor McKeag; Anne Sillars; Angus Stirling.

Wales
Aneurin Bevan University Health Board, Nevill Hall Hospital, Abergavenny, UK: Fiona Smeeton.
Aneurin Bevan University Health Board, Royal Gwent Hospital, Newport, UK: Syed Muhammad; Kofi Obuobie; Win Yin.
Cardiff & Vale University Health Board, University Hospital of Wales, Cardiff, UK: Sai Ambati; Rahim Khan; Preethi Nalla; Arshiya Tabasum.
Hywel Dda University Health Board, Glangwili General Hospital, Carmarthen, UK: Stamatios Zouras.
Hywel Dda NHS Trust, Prince Philip Hospital, Llanelli, UK: Akhila Mallipedhi.
Swansea Bay University Health Board, Singleton Hospital, Swansea, UK: Richard Chudleigh; David Williams.
